# Attentional Bias to Threat-Related Information Among Individuals With Dental Complaints: The Role of Pain Expectancy

**DOI:** 10.3389/fpsyg.2018.00786

**Published:** 2018-05-23

**Authors:** Mohsen Dehghani, Somayyeh Mohammadi, Louise Sharpe, Ali Khatibi

**Affiliations:** ^1^Department of Psychology, Shahid Beheshti University, Tehran, Iran; ^2^GF Strong Rehabilitation Centre, Department of Occupational Science and Occupational Therapy, The University of British Columbia, Vancouver, BC, Canada; ^3^School of Psychology, The University of Sydney, Sydney, NSW, Australia; ^4^Psychology Department, Bilkent University, Ankara, Turkey; ^5^Interdisciplinary Program in Neuroscience, Sabuncu Brain Research Center, Bilkent University, Ankara, Turkey; ^6^National Magnetic Resonance Research Center (UMRAM), Sabuncu Brain Research Center, Bilkent University, Ankara, Turkey

**Keywords:** attentional bias, dental pain, threat, pain expectancy, dot-probe, patients

## Abstract

Expecting pain can be perceived as a threat may involve recruitment of cognitive strategies (such as attentional avoidance) which might help the person to reduce distress. The ecological validity of the paradigms aiming to study the attentional biases toward or away from threatening stimuli by manipulating the perception of threat in experimental settings has been questioned. Therefore, the current study aims to investigate the attentional bias toward or away from the threat when a confrontation with a real threatening and painful condition would be expected (i.e., dental treatment). One hundred and twenty-seven patients referred to three dentistry clinics for a dental treatment (experiment participants) and 30 individuals with no dental complaints (control participants) completed this study. Patients were randomly allocated to a high pain expectancy (HPE: *n* = 65) or a low pain expectancy (LPE: *n* = 62) expectancy condition. All participants completed questionnaires of distress, fear of pain, and fear of dental pain. Furthermore, they participated in a dot-probe task that assessed their attention to painful faces, dental pictures, and happy faces. In addition, before the treatment, participants reported their anticipated pain intensity and after the treatment, they reported the pain intensity that they perceived during the treatment using two separate visual analog scales. Patients in the HPE group showed a bias away from dental pictures compared to LPE and control group participants. HPE group patients also reported greater pain intensity during the treatment compared to LPE patients. Greater attentional bias away from dental pictures among HPE patients was associated with higher levels of fear of pain, fear of dental pain, and stress. Avoidance of highly salient threatening images can be seen as an unhelpful emotion-regulation strategy that individuals use to manage their fears. However, in this study, avoidance was associated with poorer outcomes.

## Introduction

Cognitive-affective models consider attentional biases including attending to, sustaining attention, and attending away from threatening and distressing stimuli as detrimental components of many psychopathologies (Pine et al., 2005; Bar-Haim et al., 2007). For example, studies have shown that individuals with anxiety disorders are prone to exhibit attentional biases to threat-related stimuli (Bar-Haim et al., 2007; Barry et al., 2015). Researchers have also found attentional biases to pain-related information among individuals with chronic pain and also those with acute pain (e.g., (Haggman et al., 2010; Schoth and Liossi, 2010). Moreover, some recent studies observed the existence of these biases among parents, partners, and other family members of individuals with chronic pain (Vervoort et al., 2011, 2013b; Mohammadi et al., 2012, 2015). Despite the evidence, the existence of attentional bias to pain-related or threat-related information/stimuli has not always been supported by research. In contrast, some studies have demonstrated that individuals experiencing pain and anxiety disorders tend to avoid pain and threat-related information (e.g., Lautenbacher et al., 2010; Sharpe et al., 2014).

Attending away and avoiding from pain- or threat-related information can be an emotion regulation strategy and therefore, has a protective function (Knight et al., 2007; Vervoort et al., 2013a). That is, in short-term, attentional or behavioral avoidances can protect individuals from experiencing additional distress when they are expecting a confrontation with a painful or threatening stimulus (Vervoort et al., 2013a). This effect can be seen especially among individuals with higher levels of fear and anxiety (Vervoort et al., 2012, 2013a). Furthermore, it has been indicated that in situations when experiencing an actual threat is expected, it is more likely to observe attentional and behavioral avoidances (Wald et al., 2011). For example, people who live in war zones and expect a life-threatening danger exhibit an attentional avoidance from the information that is related to the life-threatening danger (Bar-Haim et al., 2010). Therefore, it can be concluded that individuals with high levels of fear and anxiety who are expecting to confront actual life-threatening situations tend to attend away from the threat-related information. Some studies have also shown that experiencing acute pain, expecting a painful stimulus, and even observing pain in another person is distressful and can potentially result in attentional avoidance among individuals with higher levels of fear and anxiety (Vervoort et al., 2013a). However, the above-mentioned findings have been mainly observed in experimental settings and participants did not expect to confront with an actual and unavoidable pain or threat. It is not clear whether attentional avoidance and attending away from pain-related information can also be observed in non-experimental settings when individuals are expecting to experience a potentially painful procedure.

Therefore, the current study aims to investigate the attentional biases toward and away from pain-related stimuli among individuals who are expecting to undergo a painful operation. To do so, we used a sample of individuals attending dentistry for a dental treatment. Using a brief manipulation, the participants were randomized to high pain expectancy (HPE) and low pain expectancy (LPE) pain expectancy groups. In addition, given that findings emphasize the importance of the relevance of the presented stimuli with the individuals’ main concern (Dear et al., 2011) we assessed participants attentional biases to three types of stimuli: happy faces, pain faces, and dental pictures using a modified dot-probe paradigm following the expectancy manipulation (Khatibi et al., 2009). First, it was hypothesized that participants in the HPE group would show a bias away from dental and pain stimuli and they show a bias toward happy stimuli in comparison to the LPE group and the control group. Furthermore, it was expected that the participants in the HPE group would perceive more pain during the dental treatment than the participants in the LPE group.

## Materials and Methods

### Participants

Sample size calculations based on power analyses reported in previously published studies (Khatibi et al., 2009) and pilots indicated that to acquire medium to large effect size in the comparison of two groups (interclass correlation coefficient of 0.05 and alpha equal to 5%) we need a sample bigger than 100 subjects in total. One hundred and twenty-seven patients attending three dentistry clinics in Tehran, Iran between July 2010 and December 2010 were invited to participate in this study. To be eligible, participants had to be over 18 years old, had sufficient literacy to complete the questionnaires, were able to use both hands and, had an appointment for a root canal treatment. Patients were excluded if they had any head injury in the last 3 years, have a current drug or alcohol abuse and persistent pain for more than 1 month in the past year or any other pain complaint except for dental-related pain during the last week. Control participants were matched with the patients based on age and educational level and had no dental-pain complaint (*n* = 30). These participants were approached by the researcher at several local cultural and educational institutions and were asked to take part in the study. The inclusion and exclusion criteria that have been used for patients were also used for recruiting this group, except that individuals who reported any dental or other pain during the last 3 months were excluded from the study. This study was carried out in accordance with the recommendations of the ethical committee of Azad University. The Ethical Committee of Azad University and the medical boards of the dentistry clinics approved this study and all participants gave written informed consent.

### Procedure and Pain Expectancy Manipulation

The researcher explained the aims of the study for dentists and secretaries and asked them to introduce the eligible patients. Then, the researcher approached eligible patients and explained the study. All patients who were approached agreed to participate in the study. After signing the consent form, participants completed a battery of questionnaires (section “Procedure and Pain Expectancy Manipulation”). Then, they were randomly assigned to one of two experimental groups and received information (pain expectancy manipulation) that either led to the high or low expectancy of pain during the dental treatment. The random assignment was conducted by Random Allocation Software which is a free tool for random assignment (Saghaei, 2004).

Two different pain expectancy manipulation sheets were prepared for participants in experimental groups (as follows). Participants were told that the information in these sheets was acquired from surveys that have been run previously in dental clinics on patients undergoing a similar operation as the one they are waiting for. Both sheets started with information regarding methods of dental care and then some information about the anesthetics used during dental operations were provided. Afterward, in the sheet for HPE group (*n* = 65), it had been written that surveys have shown that the anesthetics that patients receive in dental clinics only reduces their pain by 30% and most patients report considerable pain during the treatment. In contrast, in the sheet for LPE group (*n* = 62), participants were told that the anesthetics reduce pain by 90% and participants in previous studies experienced very little pain during the dental operation. Individuals in the control group received a sheet containing the first paragraph about the dental care.

After reading the pain expectancy manipulation sheet, they were given a 10-min break before being asked to complete the dot-probe task. Dot-probe task was performed in a separated room (no windows, deemed light) in the clinic where the subject was recruited and only the subjects and the experimenter were present in the room during the test. Participants in the control group also were invited to one of three clinics and the battery of questionnaires and the task were completed there. After completion of the task, participants in the control were debriefed and thanked for their participation. Once participants in experimental groups had completed the dot-probe task, they would attend their dental treatment. The dentists were blind to the group allocation.

After participants had completed their dental treatment, they were asked to report the amount of pain they experienced during the treatment on the VAS. In the end, they were debriefed and thanked for their time.

### Measures

A number of different self-report measures used in the current study.

#### Visual Analog Scale (VAS)

To assess pain intensity, the visual analogue scale (VAS) was used. The VAS is a 10-cm, ungraded horizontal line. The left anchor indicated a “minimum intensity of pain” whereas the right anchor indicated a “maximum imaginable intensity of pain.” Using two separate VAS lines, before the treatment, patients were asked to indicate the level of pain that they anticipate experiencing during the treatment (i.e., anticipated pain) and after the treatment, they were asked again to indicate the level of pain that they perceived during the treatment (i.e., perceived pain).

#### Depression, Anxiety, and Stress Scale (DASS; Lovibond and Lovibond, 1995)

To evaluate depression, anxiety, and stress among patients and participants in the control group, the DASS was used. This scale has 42 items and three subscales (i.e., depression, anxiety, and stress). The reliability and validity of DASS are well established. Lovibond and Lovibond (1995) have reported Cronbach’s alpha for each subscale as follows: anxiety = 0.84, depression = 0.91, and stress = 0.90. The DASS does not rely predominantly on somatic items and is less likely to be inflated in a chronic pain sample. Recent research has confirmed the DASS to be the instrument of choice to measure depression in chronic pain (Sarda et al., 2008; Akbari et al., 2016). In the current study, the Cronbach’s alphas of depression, anxiety, and stress subscales in patients were 0.92, 0.87, and 0.85 and in the control participants, the Cronbach’s alphas were 0.84, 0.90, and 0.89.

#### Fear of Pain Questionnaire-III (FPQ-III; McNeil and Rainwater, 1998)

The Fear of Pain questionnaire-III (FPQ-III) is a self-report questionnaire consisting of 30 items assessing fear of pain in specific situations (McNeil and Rainwater, 1998). Each item is rated on a 5-point Likert-type scale. FPQ-III has been used in both clinical (Ochsner et al., 2006) and non-clinical populations (Osman et al., 2002). In the current study, the Cronbach’s alphas of the FPQ for the patients and the control participants were 0.92 and 0.94, respectively.

#### Fear of Dental Pain Questionnaire (FDP; Van Wijk and Hoogstraten, 2003)

The Fear of Dental Pain Questionnaire (FDP) consists of 18 items and assesses the fear of pain associated with different dental treatments rated on a 5-point Likert-type scale (1 = no fear, 5 = extreme fear). The total score ranges between 18 and 90, with higher scores showing higher levels of fear of dental pain. This questionnaire was developed as the dental equivalent of the FPQ-III (Van Wijk and Hoogstraten, 2003) and has good internal consistency (alpha = 0.93) and test-retest reliability, i.e., 0.75 after 5 weeks (Van Wijk and Hoogstraten, 2003). In this study, the Cronbach’s alphas of the FDP were 0.95 in the patients and 0.96 for the control participants.

#### Manipulation Check Questionnaire

To investigate the effect of instructions about the effectiveness of the analgesic drug participants were asked to rate their anticipation of their pain level during the treatment before the treatment and also after the treatment were asked whether they believed the manipulation at the beginning of the experiment changed their expectation about the level of pain that they anticipated experiencing. All confirmed that the manipulation had changed their expectation about their anticipation of the level of pain that they would experience during the treatment.

#### Pictorial Dot-Probe Task

A pictorial version of the dot-probe task was designed for this study, based on previous versions of the dot-probe for assessing biases toward happy and painful faces (Khatibi et al., 2009). All the stimuli were presented on a 15″ DELL laptop monitor, at a viewing distance of 50 cm. A black circle was presented as a fixation point at the center of the monitor. This point remained on the screen for 200 ms and was then replaced by a pair of pictures (6^∗^4.5 cm), one above the other (5 cm away from the fixation point). The pictures remained on the screen for 300 ms. The duration of 300 ms is chosen based on Fox et al. (2002) suggestion that faster presentation of cues avoid the return of attention to the central and initial location and therefore it measures attentional engagement. After 300 ms, both pictures were disappeared, and an arrow appeared at the same location as one of the pictures. The participants were asked to press “D” (Labeled ←) when the arrow pointed to the left and press “L” (labeled →) when the arrow pointed to the right. The arrow faded away after participants pressed one of the keys. The computer recorded the participant’s response. If participants did not press any key, the arrow automatically disappeared after 1500 ms and next trial commenced automatically.

**FIGURE 1:**
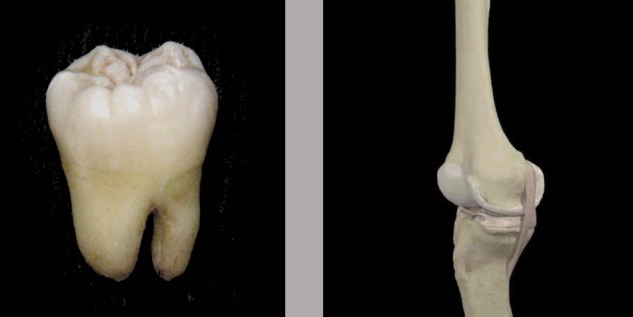
An example of a picture of a tooth (Left) and paired picture (Right) used in the dot-probe task.

There were 240 experimental trials, which consisted of 80 trials of three stimulus pairs, as follows: happy faces/neutral faces; painful faces/neutral faces; dental-related pictures/body-related pictures. The three sets of stimuli were presented in separate blocks in a different random order across participants. Photos that were used in the first two stimuli sets (happy/neutral; pain/neutral) were adopted from the study of Khatibi et al. (2009). Pictures of teeth were chosen from a wide range of related photos in the archive of the dental clinic and filtered by two expert dentists (the chosen photos did not present damaged or deformed teeth). Several pictures of different body parts that were matched by tooth pictures based on their colors, contrast, and brightness had been selected among a wide variety of pictures in other clinics’ archives (For an example of a target image and paired picture see **Figure 1**). In patients at dental clinics the tooth is a part of the body which is expected to receive a painful treatment. Therefore, dental pictures were included to test the effect of perceived threat toward a specific body location.

The task was presented in four blocks: one practice block and three experimental blocks. The practice block consisted of ten trials. The presented pictures in this block were landscape pictures and were only used in the practice trials. The experimental blocks consisted of 80 trials for happy/neutral, 80 trials for painful/neutral, 80 trials for teeth/body pictures. The order in which the blocks were presented was randomized, as was the order of presentation of each of the trials. Each picture was presented randomly in four possible combinations (i.e., target up/probe down; target up/probe up; target down/probe down; target down/probe up). Participants were asked to take a break of 2 min after finishing each experimental block. However, the researcher started the next experimental block without any break if participant indicated that he/she preferred to continue. On average, it took 12 min for each participant to complete the task.

Before conducting the analyses on the dot-probe task results, the raw reaction time data was inspected. Incorrect responses and outliers (>2.5 standard deviations, Ratcliff, 1993) constituted less than 1 and 1.9% of total trials, respectively. Therefore, we removed both from the available data and did not consider them any further. Moreover, due to technical problems (i.e., the software did not record any reaction times), some data were not available: data of 11 individuals on attention index to painful faces, data of 7 individuals on attention index to happy faces and data of four individuals on attention index to dental-related pictures.

### Statistical Plan

Because of software failure responses of several subjects during some tasks were not recorded (four subjects for teeth/body task, three subjects for the happy/neutral task and three subjects for the painful/neutral task). Since there was no subject with complete missing data none of the participants were removed from the analyses and only degrees of freedom were adjusted accordingly.

To examine potential baseline differences between the HPE, LPE, and control groups on demographic and other variables, a series of one-way ANOVA and χ^2^s were conducted.

Three indices of attentional bias were calculated for each type of stimuli: happy/neutral; pain/neutral; and dental pictures/other parts of the body) by using the following formulae (T = “Target”, P = “Probe”, U = “Up”, D = “Down”, i.e., TUPD = “target up, probe down”):

(I) Congruent Trials = (TUPU+TDPD)/2
(II) Incongruent Trials = (TUPD+TDPU)/2
(III) Bias Index = Incongruent Trials-Congruent Trials

As such, a positive score is an indication of selective bias *toward* the location of the specific target and a negative score shows attentional avoidance or attending *away from* the target picture.

Initially, two ANCOVAs were run to determine the impact of the manipulation. The major analysis to determine the effect of the threat manipulation on selective attention was a 3 (pain groups: HPE; LPE; controls) × 3 (stimuli: pain; happy; dental pictures) mixed model ANOVA. In order to interpret main and interaction effects, planned comparisons were conducted to investigate differences for the HPE group with the other two groups and to compare responses to the three types of stimuli. Further, to determine whether any biases were relative or actually different from zero, attentional biases toward different types of stimuli were compared within each group to zero using one-way *t*-tests.

## Results

### Descriptive Information

The mean age of the participants in the HPE, LPE, and the control groups were 28.78 (SD = 8.29), 30.74 (SD = 10.27), and 30.66 (8.11). According to the results of one way ANOVA there were no significant difference between the groups (i.e., HPE, LPE, and control) in age [*F*(2,154) = 0.86, *p* = 0.43], education level [*F*(2,154) = 2.03, *p* = 0.13], fear of pain [*F*(2,154) = 0.53, *p* = 0.59], fear of dental pain [*F*(2,154) = 2.27, *p* = 0.11)], depression [*F*(2,154) = 2.61, *p* = 0.08], and stress [*F*(2,154) = 2.00, *p* = 0.14]. Similarly, χ^2^s also indicate that the differences between gender (HPE: 37 females and 28 males; LPE: 31 females and 31 males; Control: 17 females and 13 males; χ^2^= 0.43, *p* = 0.47) and marital status (HPE: 32 married and 32 single; LPE: 27 married and 35 single; Control: 13 married and 17 singles; χ^2^= 0.47, *p* = 0.48) were not significant. However, there was a difference between the groups in the anxiety level [*F*(2,154) = 3.89, *p* = 0.02]. Tukey *post hoc* tests revealed that there was a significant difference between the control group and the LPE group in the level of anxiety, *p* = 0.04. Nonetheless, to be conservative, anxiety was controlled for in subsequent analyses. The mean scores and standard deviations between psychological and pain-related variables were presented in **Table 1**.

**Table 1:** Means and SDs of Psychological and pain related variables among High Pain Expectancy (HPE) group, Low Pain Expectancy (LPE) group and Control group.

	High pain expectancy group	Low pain expectancy group	Control group
Anticipated pain^∗^	54.85 (28.49)	49.22 (28.43)	–
Perceived pain^∗∗^	22.92 (25.00)	11.40 (19.01)	–
Depression	20.01 (15.67)	24.56 (14.43)	17.73 (13.63)
Anxiety	10.15 (7.67)	13.01 (7.34)	8.9 (7.05)
Stress	16.4 (9.09)	17.96 (8.33)	14.16 (8.0)
Fear of pain	83.83 (19.45)	83.11 (18.88)	79.53 (19.27)
Fear of dental pain	54.38 (15.62)	54.85 (15.33)	47.86 (16.27)

*^∗^Patients anticipation of the pain that they may experience during the treatment-reported before the treatment. ^∗∗^Patients perception of the pain that they perceived during the treatment-reported after the treatment.*

### Manipulation Check

In order to determine whether the manipulation was successful, we conducted a one-way ANCOVA on the anticipated pain level between the two dental groups (i.e., HPE and LPE) and anxiety was added as a covariate. The results of the ANCOVA demonstrated that the two groups differed significantly on the anticipated pain level [*F*(1,124) = 3.78, *p* = 0.05]. This indicates that the manipulation was successful and those in the HPE expected to experience higher pain than those in the LPE condition. A similar ANCOVA was run on the perceived pain level and demonstrated that two groups differed significantly on the perceived pain level [*F*(1,124) = 1.97, *p* < 0.01] with patients in the HPE group perceived more pain during the treatment than those in the LPE group.

### Dot-Probe Results

**FIGURE 2:**
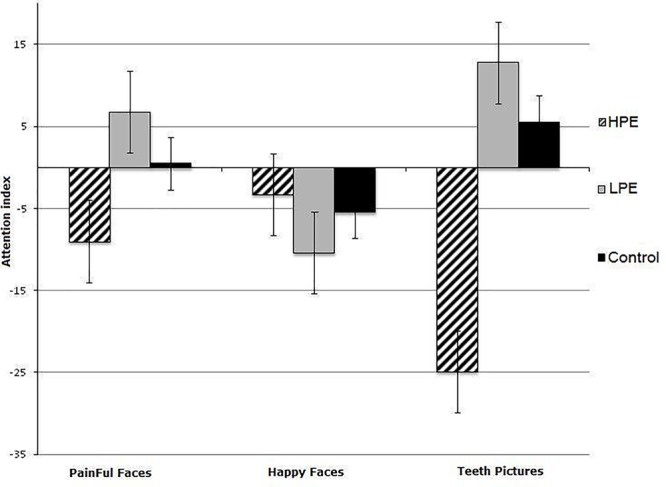
Mean Reaction times to happy, pain, and teeth related pictures in High Pain Expectancy (HPE), Low Pain Expectancy (LPE), and control groups.

Attentional bias indices were analyzed using a mixed model ANCOVA with stimuli (3: happy, pain, and dental targets) as the within-subject factor and groups (3: HPE, LPE, and Control) as the between-subjects factor (See **Figure 2**). There was no significant main effect of stimuli [*F*(2,284) = 0.46, *p* = 0.63] but there was a significant interaction between the group and the stimuli [*F*(4,280) = 4.953, *p* = 0.001] which could be explained by the significant main effect of group [*F*(2,140) = 8.74, *p* < .001]. Follow-up analyses by comparing attention indices in three groups indicated that for the control group there were no significant differences between attention indices for pain, happy and teeth blocks (*p*s > 0.49). For HPE group there was no difference between attention indices for pain vs. happy (*p* = 0.42) while there were significant differences between attention indices for pain vs. teeth and happy vs. teeth (*p*s < 0.003). For LPE group there was no significant difference between indices for pain vs. teeth but there were significant differences between indices for happy vs. pain and happy vs. teeth (*p*s < 0.02).

To determine the direction of any attentional biases, the three groups’ attentional biases to each of the three categories of stimuli were compared to zero with one-way *t*-tests. The results showed that the participants in the HPE group did not show biases toward either happy [*t*(61) = 0.881, *p* = 0.382] or painful faces [*t*(59) = 1.625, *p* = 0.11]. However, they did attend away from dental pictures [*t*(61) = 8.027, *p* < 0.001]. In contrast, those in the LPE group attended away from happy faces [*t*(57) = 2.046, *p* = 0.045] and toward dental pictures [*t*(61) = 3.181, *p* = 0.002], but not toward or away from pain faces [*t*(55) = 1.605, *p* = 0.114]. However, For the participants in the control group, the biases toward happy faces [*t*(30) = 0.523, *p* = 0.605], painful faces [*t*(30) = 0.051, *p* = 0.960] and dental pictures [*t*(30) = 0.642, *p* = 0.526] all did not differ from zero. **Table 2** provides an overview of mean RTs (and standard deviations) for the congruent and incongruent trials during three different dot-probe tasks and between the three groups of participants and **Table 3** presents the means and standard deviation of attention indices for the three dot-probe tasks in each group.

**Table 2:** Mean and SD of reaction times to congruent and incongruent trials during three dot-probe tasks for High Pain Expectancy group, Low Pain Expectancy group, and Control group.

			High pain expectancy group	Low pain expectancy group	Control group
Happy-neutral task	Congruent	Mean	531.64	491.47	524.27
		SD	207.31	190	168.89
	Incongruent	Mean	528.36	481.05	518.83
		SD	202.73	190.81	168.39
Painful-neutral task	Congruent	Mean	459.9	537.67	505.29
		SD	205.28	170.77	193.97
	Incongruent	Mean	450.88	544.49	505.8
		SD	202.27	166.41	194.14
Teeth-body task	Congruent	Mean	493.5	527.71	493.77
		SD	212.56	174.05	222.37
	Incongruent	Mean	468.57	540.57	499.35
		SD	201.18	170.44	211.39

**Table 3:** Means and SDs of attention index among High Pain Expectancy group, Low Pain Expectancy group, and Control group.

	High pain expectancy group	Low pain expectancy group	Control group
Attention to pain stimuli	–9.01 (42.96)	6.81 (31.79)	0.51 (54.98)
Attention to happy stimuli	–3.27 (29.29)	–10.42 (38.78)	–5.44 (56.97)
Attention to teeth stimuli	–24.93 (24.32)	12.84 (31.80)	5.58 (46.87)

*Each cell represents mean (SD).*

Finally, bivariate Pearson product-moment correlations were conducted to examine the relationships between attentional indices and other psychological variables in the study. The correlation analyses were conducted for each group separately. However, there was no significant correlation between attention indices and psychological variables in the LPE and the control groups, hence only the results of the correlations in the HPE group are presented in **Table 4**. As **Table 4** shows anticipating more pain was associated with being more likely to avoid painful faces (*r* = -0.27) and dental pictures (*r* = -0.27). The attentional biases toward dental pictures and painful faces were correlated (*r* = 0.34). However, attentional biases toward painful faces were not associated with any of the psychological variables, except for anticipated pain. In contrast, biases away from dental pictures were associated with greater fear of pain (*r* = -0.39), more stress (*r* = -0.28) and greater fear of dental pain (*r* = -0.26). Importantly, pain experience during the dental treatment was not associated with attentional biases.

**Table 4:** Correlations between pain-related variables and DASS scales among pain expectancy group.

	1	2	3	4	5	6	7	8	9
(1) Teeth index	1								
(2) Pain index	0.339**	1							
(3) Happy index	–0.135	0.006	1						
(4) Fear of pain	–0.395**	–0.314*	0.027	1					
(5) Fear of dental pain	–0.256*	–0.218	0.151	0.728**	1				
(6) Depression	–0.176	–0.145	0.155	0.330**	0.423**	1			
(7) Anxiety	–0.223	–0.12	0.185	0.356**	0.472**	0.957**	1		
(8) Stress	–0.280*	–0.072	0.312*	0.352**	0.504**	0.730**	0.784**	1	
(9) Anticipated pain^∗^	–0.280*	–0.273*	0.184	0.587**	0.755**	0.405**	0.420**	0.446**	1
(10) Perceived pain^∗∗^	–0.096	0.006	0.216	0.137	0.357**	0.264*	0.259*	0.236	0.475**

*^∗^p* < *0.05; ^∗∗^p* < *0.01. ^∗^Patients anticipation of the pain that they may experience during the treatment-reported before the treatment. ^∗∗^Patients perception of the pain that they perceived during the treatment-reported after the treatment. DASS, Depression, Anxiety, and Stress Scale.*

## Discussion

The aim of this study was to investigate the impact of pain expectancy on attentional biases toward pain-related stimuli, as well as perceived pain during the dental treatment. We hypothesized that those in the HPE group would experience biases away from dental pictures and painful faces and show biases toward happy faces in comparison with the participants in the LPE and control groups. Moreover, we expected to observe a difference between the participants in the two pain expectancy groups in the level of pain that they perceived during the dental treatment.

Our results showed that the pain expectancy manipulation was effective in creating a small, but significant difference in pain expectation level between the HPE and the LPE groups. That is, those in the HPE group expected higher levels of pain compared to those in the LPE group. In the main analyses, we found a significant main effect of group, which was informed by a significant interaction between group and stimuli. Follow-up analyses revealed that this effect was largely due to the attentional bias responses to dental pictures. That is, participants in the HPE group had an attentional bias away from dental pictures. In contrast, participants in the LPE group demonstrated an attentional bias toward dental pictures but away from happy faces. In addition, those who were led to expect higher levels of pain actually reported higher levels of perceived pain during the dental procedure. Although biases away from dental pictures and painful faces were associated with anticipated pain in the HPE group, they were not associated with the level of perceived pain during the dental treatment. However, those in the HPE group who were high in fear of both dental and general pain were more likely to show avoidance of dental pictures, although there was no association between attentional biases and the perceived pain during the dental treatment.

These results add to the growing body of literature that confirms that attentional biases in pain are “more complex than originally thought” (Sharpe et al., 2014). Firstly, these results indicate that the specificity of stimuli is an important determinant of attentional biases. In this study, where both experimental groups were about to undergo a painful dental treatment, it was the attentional biases toward dental pictures (rather than painful faces) that differentiated the groups. The specificity of biases has previously been demonstrated in other settings and it has been now well established that chronic pain patients demonstrate biases toward sensory pain words and not other types of pain words (Dehghani et al., 2003; Haggman et al., 2010). Further, it is demonstrated that biases often observed to idiosyncratically selected stimuli (Dear et al., 2011). These results emphasize the importance of the specificity of the stimuli in attention bias research.

Secondly, these results indicate that expecting a confrontation with painful and threatening stimuli is related to how an individual attend or avoid specific stimuli. Presumably, for all the experimental participants who were about to undergo a painful dental treatment, there was some expectation of pain. Pain, by its nature, is inherently threatening (Williams, 2002). Hence, by manipulating the pain expectancy of a dental treatment, the threat associated with that procedure was also manipulated. Our results show that those individuals who faced with the threat of a dental treatment but expected a good pain relief (i.e., a moderate level of threat) demonstrated a bias toward dental pictures, whereas when that threat is further increased, avoidance of dental pictures was observed. Relatively little research has directly assessed the impact of threat on attentional biases. Boston and Sharpe (2005) found that, under threatening conditions, individuals showed a relative bias toward affective pain words in comparison to sensory pain words, whereas the opposite was true under low threatening conditions. More recently, Sharpe et al. (2017) found that under conditions of high threat, those high in fear of pain were avoidant of affective pain stimuli, whereas for those low in fear of pain but high in threat (i.e., a moderate level of threat) difficulty disengaging was observed. In the current study, the dental treatment was considerably more threatening than the cold pressor used in the Sharpe’s study, which likely explains why the threat was higher and therefore avoidance of the most salient stimuli rather than vigilance was observed.

The vigilance-avoidance hypothesis argues that as the threat level increases, people become more vigilant to stimuli (i.e., notice it more quickly). However, the pattern for sustained attention is different. As threat increases from low levels, people are thought to attend more to salient stimuli. However, when the level of threat increases, the tendency to over-attend switches to avoidance (Todd et al., 2015). The results of this study are consistent with this hypothesis. Furthermore, some of the eye tracking studies confirm a similar pattern. Yang et al. (2012) found that chronic pain patients were more likely to direct the initial focus of attention to pain-related stimuli, but this was followed by shorter fixations, indicating an avoidance. Liossi et al. (2011) replicated the findings of earlier studies, and although they failed to see significant differences between individual suffering from chronic daily headache and healthy controls’ attentional bias to scores for shorter stimulus presentations (500 ms), found a trend to indicate subsequent avoidance. Again, our results are consistent with these studies.

In this study, we employed reaction time rather than eye gaze behavior. Hence, the reaction time results indicate that at 300 ms, avoidance rather than vigilance characterized the participants’ responses when HPE existed. However, this is only a snapshot of attention at this time point. It is possible, as Yang et al. (2013) reported that this avoidance is characterized by a pattern of an early disengagement but subsequent a re-engagement. Further research is needed, using eye tracking to determine the precise nature of these biases.

Importantly, Yang et al. (2012) also found that similar attentional patterns (i.e., vigilance-avoidance) were more evident in healthy people with high fear of pain. In this study, we also found an association between fear of pain and, specifically fear of dental pain and attentional biases. Consistent with Yang et al. (2012), those with higher levels of fear of pain were more likely to avoid dental pictures when expecting higher levels of pain. In contrast, in people with no chronic pain, Vervoort et al. (2013a) found that catastrophizing (usually associated with fear of pain) predicted early avoidance, particularly when stimuli (facial expressions) represented higher levels of pain. These results also suggest that as threat increases (in this case of the stimuli), vigilance is replaced by avoidance.

While attentional biases in this study were associated with the anticipated pain, they were not associated with the perceived pain during the dental treatment. We had anticipated that pain would be associated with the attentional biases toward happy faces and also associated with the attentional biases away from dental pictures and painful faces (Lautenbacher et al., 2010; Sharpe et al., 2014). However, this hypothesis was not confirmed. Hence, we cannot conclude that the attentional biases induced by the manipulation were associated with the perceived pain. Rather, it was the anticipated level of pain that was related to the observed attentional biases. Indeed, despite the fact that the differences in the anticipated pain were small, these small but significant differences had a considerable impact on attentional patterns.

Despite attention to the methodology, there were limitations of this study. Firstly, as indicated above, our measure of attentional biases relied on reaction times rather than the more direct measurement of eye gaze behavior. This provides only a snapshot of attention in one point of time, and the reliability of this approach has been questioned (Dear et al., 2011). Further, we did not explicitly measure pain-related threat, therefore our explanation that moderate levels of threat are associated with biases toward salient stimuli, whereas higher levels of threat are associated with avoidance remains speculative. Further, we did not measure biases before and after the manipulation for practical reasons and therefore we cannot exclude the possibility that these differences were pre-existing. However, the lack of other differences between the two experimental groups makes this interpretation less likely.

Despite these limitations, this study used an experimental manipulation regarding pain expectancy and examined attentional biases in a clinical population. The fact that we were able to demonstrate clear differences in the way that the HPE group responded in comparison to the LPE group is important. There have been a number of attempts to manipulate attentional biases to pain, using a variant of the dot-probe task, where participants are trained to attend away from pain-related stimuli with positive results (McGowan et al., 2009; Sharpe et al., 2012). The assumption of these studies is that attention toward pain is unhelpful, and therefore training people away from pain should have therapeutic effects. However, more recent research has suggested that the direction of the training may be dependent, in part, on the stimuli. Todd et al. (2016) found that when training with affective pain words (i.e., those which are the most emotionally salient and which have been implicated in avoidance), training toward rather than away from affective pain words was associated with better pain outcomes. This study and other recent studies demonstrating the importance of avoidance (e.g., Sharpe et al., 2017) suggest that the conditions under which the training occurs may not only determine what stimuli might be targeted, but also whether attention should be trained toward or away from the stimuli. These results provide another piece of this puzzle, suggesting that at least under conditions of high threat, avoidance rather than vigilance might explain attentional bias to pain-related information.

## Author Contributions

MD and AK were involved in the study design, the data acquisition, analyses, and the manuscript preparation. SM and LS were involved in analyses and the manuscript preparation. All authors read and approved the final version of the manuscript.

## Conflict of Interest Statement

The authors declare that the research was conducted in the absence of any commercial or financial relationships that could be construed as a potential conflict of interest.
